# Primary prostatic urothelial carcinoma presenting as persistent perirectal abscess

**DOI:** 10.1016/j.eucr.2026.103459

**Published:** 2026-04-21

**Authors:** Emily F. Yamashita, Ming-Yeah Y. Hu, John Yablonski, Andrew C. Peterson

**Affiliations:** a Department of Urology, Duke University, Durham, NC, United States; b Department of Pathology, Duke University, Durham, NC, United States

**Keywords:** Urothelial cell carcinoma, Urologic oncology, Perirectal abscess, Cystourethrogram

## Abstract

Primary prostatic urothelial carcinoma is a rare, often asymptomatic, disease. A 62-year-old male presented with chronic, clear fluid leakage from a perirectal abscess and recurrent pelvic fluid collections involving the prostate, penis, scrotum, and perirectal space. No fistulas were identified on surgical exploration and cystogram, and no masses or lesions were seen on CT or cystourethroscopy. Delayed presentation of pelvic lymph node metastatic disease led to the diagnosis of primary prostatic urothelial carcinoma. This is a compelling and educational case that emphasizes the need for oncologic vigilance in patients with persistent, unexplained pelvic or perineal fluid collections and fistula.

## 1 Introduction

Prostatic involvement is common in patients with primary bladder urothelial carcinoma.^1^ However, primary prostatic urothelial carcinoma (UCP) is rare, comprising 1% to 4% of prostatic cancers.^2^ While often difficult to diagnose early, the common presentations of primary UCP include obstructive symptoms, hematuria, and irritative symptoms resembling prostatitis.^3^, ^4^, ^5^

We present a case of a male who presented with a persistent perirectal fistula, recurrent pelvic fluid collections and abscesses, urethral stricture disease, negative computed tomography scans and pelvic MRI, and multiple surgical and endoscopic examinations who was subsequently diagnosed with delayed development of locally advanced metastatic urothelial carcinoma of prostatic origin.

## 2 Case presentation

**Fig. 1:**
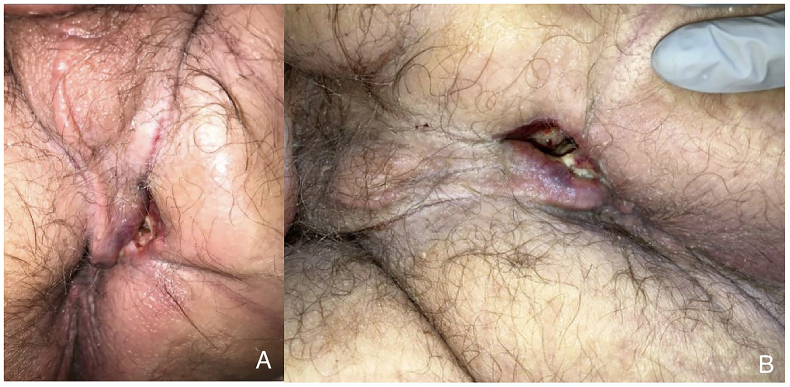
A) Chronic perirectal abscess post-incision and drainage, complicated by recurrent infection. B) Persistent perirectal abscess after a two-week course of antibiotics.

**Fig. 2:**
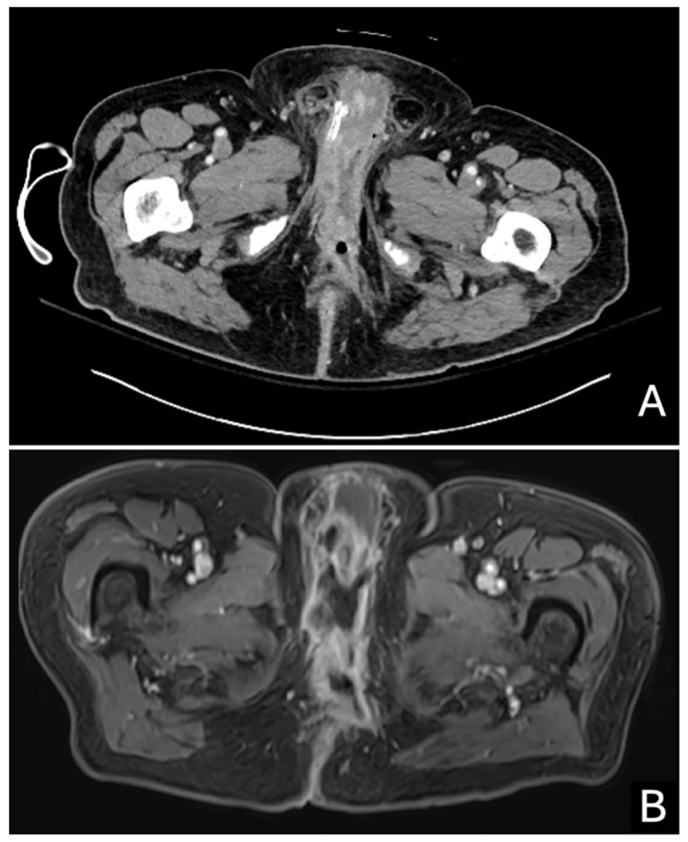
Imaging of the fluid collections. A) CT of scrotal fluid extending to the perineum, measuring 2.2 × 4.6 cm. B) MRI of fluid collection in perirectal space extending to penile base.

A 62-year-old male with a past medical history of uncontrolled type 2 diabetes, hypertension, atrial flutter, tobacco use, and prior horseshoe perirectal abscess with anorectal fistulization treated twenty years ago presented to the emergency department at another facility for shortness of breath and was admitted for non-ST elevated myocardial infarction. Simultaneously, he was found to have continued clear drainage from a perirectal abscess that was drained three months prior and treated with antibiotics (Fig. 1). He had reported multiple prior procedures over recent years for his history of anorectal fistula and abscess but records were incomplete. Computed tomography (CT) at presentation revealed a fluid collection involving the left prostate and left mesorectal space (Fig. 2). Foley catheter placement to divert urine away from the presumed urethral fistulous tract required serial dilation at that time. CT cystogram and surgical exploration at that presentation revealed no fistula or extravasation from the bladder. The patient was discharged on antibiotics and did not present for follow-up.

Two years later, the patient re-presented to multiple other facilities with persistent weakness, chills, and suspected abscess/infection of the scrotum, penis, and perianal region. Again, urology was consulted for urethral stricture disease and difficult Foley catheter placement requiring dilation. He had evidence of urethral trauma on cystoscopy at that time. He was pending further exam under anesthesia and operative exploration after antibiotic treatment but follow-up was not completed.

**Fig. 3:**
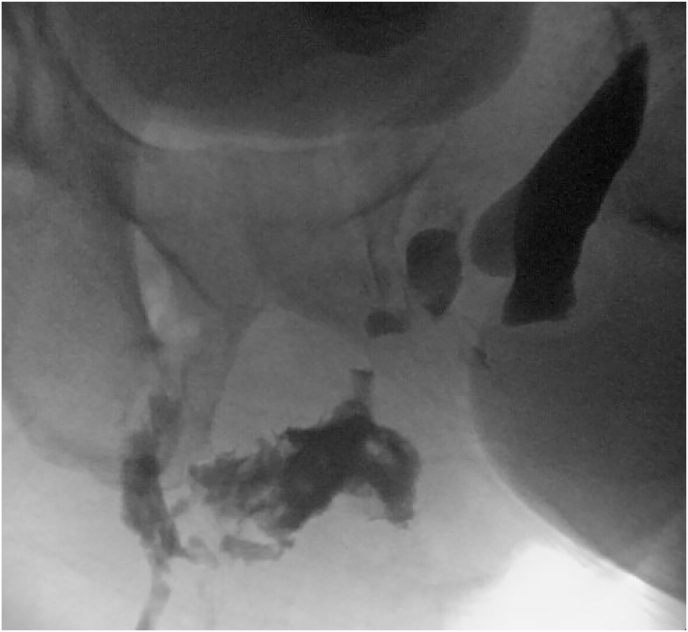
Cystourethrogram showing pan-urethral stricture with extravasation of contrast from bulbar urethra into posterior cavity.

Two months later, the patient presented to our facility for the first time and was readmitted five times for recurrent fluid collections and abscesses involving the scrotum, penis base, perineum, prostate, perirectal space, bladder, and anterior pelvic wall. He was co-managed by urology and colorectal surgery given his prior known anorectal fistula. He was managed with multiple operative drainages, exploration, cystoscopies, and anoscopies. Cystourethroscopy and retrograde urethrogram revealed panurethral stricture with prostatic urethra erosion into an abscess cavity (Fig. 3). There were no obvious masses or lesions in the urethra and bladder. The prior fistulous opening appeared healed on anoscopy. Initial scrotal and rectal biopsy was benign with necrosis and benign colonic tissue, respectively. Tissue culture was positive for mixed gram-positive organisms. Additional work-up included normal prostate specific antigen (PSA) of 1.49. On CT, his prostate volume was 30 mL and notable only for nonspecific calcifications presumed to be secondary to prior prostatitis. Urinary diversion was initially managed with dilation and foley placement, but ultimately the patient underwent suprapubic catheter placement.

Persistent fluid collections and infections prompted pelvic MRI, which showed no evidence of urethral or rectal connection to the abscesses. However, delayed phase contrasted CT and repeat cystogram with the suprapubic tube capped confirmed urinary source of the complex fluid collections. He was maximally diverted with bilateral PCNs in addition to his suprapubic tube and drains. He was referred for further workup for with rheumatology and urologic oncology to evaluate for autoimmune or malignant causes of significant urethral pathology of unknown etiology.

**Fig. 4:**
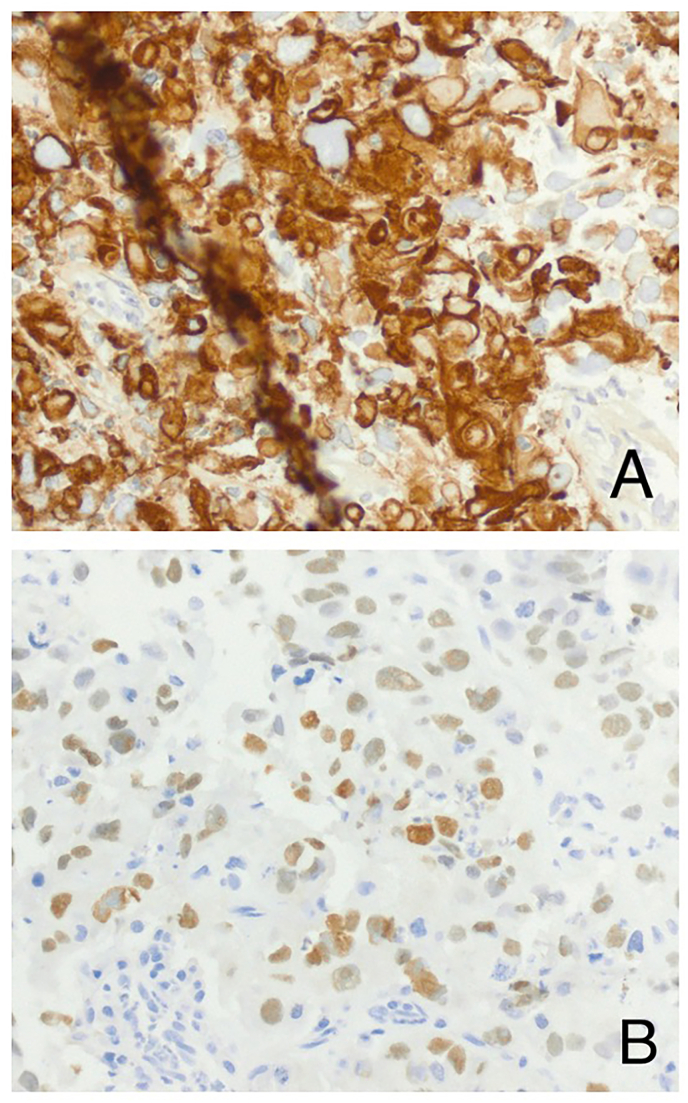
Core needle biopsy of left inguinal lymph node. A) Tumor cells staining positive for Pan-CK, consistent with epithelial origin. B) Tumor cell nuclei staining patchy positive for GATA-3, supporting urothelial origin.

Finally, a subsequent CT during another ED presentation (6 months after initial presentation to our facility) demonstrated increasing, heterogenous pelvic lymphadenopathy. Lymph node biopsy revealed urothelial cell carcinoma, presumed to be of prostatic origin (Fig. 4). The patient was diagnosed with cT4N+M0 urothelial cell carcinoma and started on gemcitabine cisplatin treatment. One cycle of treatment reduced the tumor size; however, imaging revealed a necrotic core with superimposed infection. Despite multiple rounds of different antibiotics, the patient had persistent scrotal infections. Incision and drainage was not performed because it would not provide source control. The patient decided to stop treatment and pursue comfort care.

## 3 Discussion

Primary UCP is a rare malignancy, and the literature remains primarily limited to case reports. Diagnosis is challenging because patients often lack symptoms and have normal PSA levels.^4^^,^^6^ Digital rectal examination is frequently normal until advanced stage disease.^4^ Patients with symptoms commonly present with obstructive voiding patterns, dysuria, and hematuria.^3^, ^4^, ^5^ Primary UCP is typically aggressive and survival is poor.^3^, ^4^, ^5^ Other prostatic malignancies include adenocarcinoma and squamous cell carcinoma. Adenocarcinoma is the most common prostatic malignancy, while squamous cell carcinoma frequently develops in the setting of chronic inflammation. Although our patient had evidence of chronic inflammation, biopsy revealed primary UCP, demonstrating the diagnostic complexity of the case.

Our case highlights a unique presentation of primary UCP that has not yet been described in the literature. Our patient had a several-year history of recurrent pelvic fluid collections and urethral pathology of unknown etiology. The original working diagnosis was an anorectal source given his known prior diagnosis of urethral trauma. Malignancy was ultimately detected after metastatic spread to the pelvic lymph nodes was observed on CT. Primary UCP in our patient caused partial urinary obstruction and chronic inflammation, which led to urinary extravasation into surrounding tissue. This manifested in our patient's initial presentation of fluid collections and perirectal abscess.

While the patient was eventually referred to urologic oncology to workup malignant causes of his significant urethral pathology, earlier suspicion could have prevented delayed diagnosis. Repeat imaging negative for masses, absence of lesions on cystourethroscopy, and history of urethral trauma and prior anorectal fistula led to initial low suspicion for malignancy in our patient, which delayed prostate-, urethral, and deep-tissue biopsies that may have aided in earlier diagnosis. CTs consistently showed fluid collections, wall thickening, edema, soft tissue gas, and fat stranding. Rectal biopsy and tissue culture of his abscesses revealed infection as a source of his inflammation. Although infection contributed to our patient's complex presentation, malignancy was the underlying source of our patient's persistent fluid collections, demonstrating the need for oncologic vigilance in such patients. Additionally, this case emphasized the need to have a higher suspicion for a rare underlying cause in a patient presenting with a persistent, complex problem for multiple years. While patient presentation at multiple facilities, lack of records, and difficulty in maintaining follow up can hinder diagnosis, this is a reminder that clinicians should take extra care in putting the entire story together and expand the differential diagnosis.

## 4 Conclusion

Primary UCP is rare and often asymptomatic until advanced stages, leading to diagnostic challenges. It should be considered in patients with unusual urinary fistulas and urethral stricture disease, even in those with negative imaging and cystourethrograms. This case emphasizes the need for oncologic vigilance in patients with persistent, unexplained pelvic or perineal fluid collections and urethral pathology of seemingly unknown etiology.

## CRediT authorship contribution statement

**Emily F. Yamashita:** Writing – review & editing, Writing – original draft, Visualization, Data curation. **Ming-Yeah Y. Hu:** Writing – review & editing, Conceptualization. **John Yablonski:** Writing – original draft, Data curation. **Andrew C. Peterson:** Writing – review & editing, Writing – original draft, Supervision, Conceptualization.

## Conflicts of interest

None.
